# Transcriptomic and Histopathological Effects of Bifenthrin to the Brain of Juvenile Rainbow Trout (*Oncorhynchus mykiss*)

**DOI:** 10.3390/toxics9030048

**Published:** 2021-03-05

**Authors:** Jason T. Magnuson, Kara E. Huff Hartz, Corie A. Fulton, Michael J. Lydy, Daniel Schlenk

**Affiliations:** 1 Department of Environmental Sciences, University of California, Riverside, 2460A Geology, Riverside, CA 92521, USA; daniel.schlenk@ucr.edu; 2 Department of Zoology, Center for Fisheries, Aquaculture and Aquatic Sciences, Southern Illinois University, Carbondale, IL 62901, USA; khuffhar@siu.edu (K.E.H.H.); corie.fulton@gmail.com (C.A.F.); mlydy@siu.edu (M.J.L.); 3 Institute of Environmental Health, College of Environmental and Resource Sciences, Zhejiang University, Hangzhou 310058, China

**Keywords:** pyrethroid, bifenthrin, salmonid, transcriptomics, apoptosis

## Abstract

The increased global use of pyrethroids raises concern for non-target aquatic species. Bifenthrin, among the most predominantly detected pyrethroids in the environment, is frequently measured in water samples above concentrations reported to induce neuroendocrine and neurotoxic effects to several threatened and endangered fish species, such as the Chinook salmon and steelhead trout. To better characterize the neurotoxic effect of bifenthrin to salmonids, rainbow trout were treated with environmentally relevant concentrations of bifenthrin (15 and 30 ng/L) for two weeks and assessed for changes in transcriptomic profiles and histopathological alterations. The top bioinformatic pathways predicted to be impaired in bifenthrin-exposed trout were involved in gonadotropin releasing hormone signaling, the dysregulation of iron homeostasis, reduced extracellular matrix stability and adhesion, and cell death. Subsequent histopathological analysis showed a significant increase in TUNEL positive cells in the cerebellum and optic tectum of bifenthrin-treated trout, relative to controls (*p* < 0.05). These findings suggest that low, ng/L concentrations of bifenthrin are capable of dysregulating proper neuroendocrine function, impair the structural integrity of the extracellular matrix and cell signaling pathways in the brain, and induce apoptosis in neurons of juvenile salmonids following bifenthrin treatment, which is consistent with metabolomic profiles demonstrating a common target and mechanism.

## 1. Introduction

The widespread application of pyrethroid insecticides for agricultural and urban use within the last 20 years, largely in response to the phase-out of organochlorine and organophosphate insecticides [1], has raised concern for non-target aquatic species. This shift has increased the amount of pyrethroid active compounds applied worldwide, exceeding 8000 tons in 2014 [2]. Pyrethroids are currently ranked as the third most applied insecticide group [3], with China and the United States represented as the top global producers and users of pyrethroids [4]. Though the mammalian toxicity of pyrethroids is lower than organochlorines and organophosphates [5], the overall environmental risk of pyrethroids has been suggested to be greater than these older-generation insecticides [6]. Pyrethroids are comprised of Type I and Type II chemical classes. Type I pyrethroids include permethrin, allethrin, resmethrin, and bifenthrin and lack an α-cyano group, whereas type II classes include cypermethrin, deltamethrin, esfenvalerate, and lambda-cyhalothrin and carry an α-cyano moiety.

The type I pyrethroid, bifenthrin, was the most frequently detected pyrethroid in sediment samples worldwide [3]. Bifenthrin was present in 78% of sediment samples collected from the United States, Australia, and China [3], and was recently detected in 77% of samples collected throughout California [7], with measured concentrations among the highest in the United States, 744 ng/g dry weight [3,8]. Similarly, bifenthrin was the most frequently detected pyrethroid in water samples in California, being detected in up to 79% of samples within the Sacramento-San Joaquin Delta [9,10], with the highest frequency and concentration measured from urban runoff areas (106 ng/L) [10] and following stormwater runoff events (133 ng/L) [9]. Concentrations of bifenthrin have also been measured above 3 µg/L within the Delta [11].

The Sacramento-San Joaquin Delta is an important spawning ground for endangered and threatened fish species, such as the steelhead trout (*Oncorhynchus mykiss*) and Chinook salmon (*Oncorhynchus tshawytscha*), which are found within the Sacramento and San Joaquin Rivers and associated tributaries. However, over the last few decades, large decreases in several pelagic fish species, known as the pelagic organism decline (POD), have occurred within the Delta. These decreases are most likely due to multiple stressors, such as flow regulations, habitat alteration, invasive species, human-made structures, overfishing, and increases in runoff pollution [12,13]. Subsequently, the increased use of pyrethroid insecticides, such as bifenthrin, since the mid-2000′s, coincided with decreased migratory returns in these spawning waters and may also be contributing to the POD [14,15,16,17,18]. Concentrations of bifenthrin in water samples collected from the Delta have been detected above acute and chronic benchmark values for several aquatic invertebrate and fish species (>1.3 and >40 ng/L, respectively) [10,19], and well above levels reported to dysregulate neuroendocrine function (≤1.5 µg/L) [20,21,22] and induce neurotoxicity in salmonids (≤120 ng/L) [23,24].

The primary mode of action for bifenthrin is binding to and disrupting voltage-gated Na^+^ channels, impairing neuronal function by altering the release of neurotransmitters [25,26,27]. Bifenthrin is a known endocrine disruptor in fish [28,29,30,31,32], dysregulating the hypothalamic-pituitary-gonadal (HPG) and hypothalamic-pituitary-thyroid (HPT) axis in salmonids by acting upon the dopaminergic pathway [20,21,22,31]. Bifenthrin is also capable of dysregulating Ca^2+^ homeostasis by impairing signaling molecules, as recently reported in inland silversides (*Menidia beryllina*) and zebrafish (*Danio rerio*) following bifenthrin treatment (<10 ng/L) [33,34]. Additionally, by utilizing a non-targeted metabolomics approach, we recently reported that bifenthrin can induce neuronal apoptotic, necrotic, and inflammatory responses in the brains of Chinook salmon (*Oncorhynchus tshawytscha*) [24] and steelhead trout (*Oncorhynchus mykiss*) [23] following exposure to ≤1.5 µg/L and ≤120 ng/L bifenthrin, respectively.

The use of transcriptomic profiling to better understand the underlying effects of contaminants found in aquatic systems has increased in the last decade due to reduced cost, high-through-put capabilities, and comparability between species and contaminant effects. RNA sequencing, combined with downstream bioinformatic functional annotation software, provides a platform for relating gene expression changes to predicted ontological changes, diseases, and associated modes of actions driving these responses. The goal of the current study was to characterize the neurotoxic effect of bifenthrin to a salmonid species, rainbow trout, through the use of transcriptomics and relate these molecular-level effects to histopathological changes in the brain. We hypothesized that there would be a dose dependent relationship between transcriptomic level changes and histological insult that were due to alterations in genes involved with reactive oxygen species production, inflammatory and oxidative stress responses, and apoptosis, as metabolites involved in these pathways were previously dysregulated in steelhead trout and Chinook salmon exposed to bifenthrin.

## 2. Materials and Methods

### 2.1. Exposure Treatments

Juvenile rainbow trout used for this study (*n* = 24) were obtained from Jess Ranch Lakes Fish Hatchery (Apple Valley, CA, USA) and were maintained in a 530 L flow-through Living Stream System (LS-700; Frigid Units, Toledo, OH, USA) at 12 °C for three months under a 14:10 h light:dark photoperiod. During this period, trout were fed Oncor Fry pellets (Skretting, Tooele, UT, USA) ad libitum daily. Individual rainbow trout were randomly transferred into 8 L glass aquaria and allowed to acclimate to the new tanks for 3 days before experimentation, as previously described [23]. Fish were fed Oncor Fry trout pellets every other day during exposures, which equated to 1% of their body weight. Rainbow trout (mean length = 21.5 ± 1.5 cm; mean weight = 78.68 ± 13.90 g) were exposed for a two-week period to one of three treatments: control (0.01% ethanol), 15, or 30 ng/L bifenthrin (purity > 96%, racemic mix of isomers; Chem Service, West Chester, PA, USA), which represent environmentally relevant concentrations commonly detected within the Delta during dry conditions. Fifty-percent static renewals were conducted daily. There was a total of eight replicates per each treatment group, with one fish per tank. Water samples were periodically collected throughout the waterborne exposures to measure bifenthrin concentrations.

Following a two-week waterborne exposure, juvenile rainbow trout were euthanized in sodium bicarbonate buffered MS-222 (Sigma-Alrich, St. Louis, MO, USA). Total length (cm) and weight (g) were taken and whole brains were extracted and either flash frozen in liquid nitrogen and stored at −80 °C for downstream RNA sequencing analysis (*n* = 4 per treatment group) or placed in Bouin’s solution (*n* = 4 per treatment group; Sigma-Alrich, St. Louis, MO, USA) for histological assessment. The current experiment was performed ethically and in accordance with protocol #20130010 approved by the University of California, Riverside Institutional Animal Care and Use Committee.

### 2.2. Bifenthrin Extractions and Analysis

Water samples collected (in duplicate, 1 L samples) from exposure tanks after 24 hours (h), 7 days (d), and 14 d were stored at 4 °C in amber glass bottles and extracted within two weeks following initial sample collection by liquid-liquid extraction. Bifenthrin extractions were conducted as previously reported [22]. Samples were analyzed using an Agilent gas chromatograph (6850A) and mass spectrometer (5975C; Santa Clara, CA, USA) in methane negative ionization mode. Samples were injected (2 µL) in pulsed (3.4 × 10^5^ Pa) splitless mode at 260 °C onto an HP5 MS column (30 m × 250 μm, 0.25 μm). Compounds were separated using helium carrier gas (1 mL/min, constant flow) and the following oven program: 50 °C initial temperature, ramp to 200 °C at 20 °C/min, followed by a ramp to 295 °C at 10 °C/min, with 10 min hold. Selected ion detection mode was used and the MS temperatures were: 300 °C transferline, 150 °C source, and 150 °C quadrupole. Chemstation software (Santa Clara, CA, USA; version F.01.03.2357) was used to identify and quantify the samples. The responses were calibrated using standards (0.5, 1.0, 2.0, 5.0, 10, 50, 100, 150, 200, 250 ng/mL in 9:1 hexane:acetone) fit to a quadratic equation (r^2^ > 0.999 for bifenthrin and recovery surrogate decachlorobiphenyl (DCBP)) using internal standard quantification (^13^C_12_-DCBP and D_6_-bifenthrin). A calibration curve was assessed using a 50 ng/mL standard once every eight samples (<10% difference). DCBP and bifenthrin were positively identified by retention time (<0.1% difference in comparison to standard) and by the peak area ratios of the quantification and qualifier ion (498 and 500 *m*/*z* for DCBP, 386, 387, and 241 *m*/*z* for bifenthrin, <20% difference in comparison to standard). The MDL for bifenthrin was 0.5 ng/L and reporting limit was 2 ng/L.

### 2.3. RNA Isolation, Preparation, and Sequencing

Juvenile rainbow trout brains (*n* = 4 per exposure treatment) were flash frozen and stored at −80 °C until RNA extractions. A tissue homogenizer (Omni International, Kennesaw, GA, USA) was used to homogenize whole brain samples and total RNA extracted with an RNeasy Mini Kit (Qiagen, Valencia, CA, USA). The RNA concentrations were determined using a Nanodrop-2000c (Thermo Fisher Scientific, Waltham, MA, USA) with RNA quality and integrity assessed on an Agilent 2100 Bioanalyzer chip. All samples used for downstream library preparation had high-quality RNA Integrity Numbers (RIN > 9). RNA was diluted to 1 μg per sample, placed on dry ice, and sent to Novogene (Sacramento, CA, USA) for library preparation and sequencing analysis. Sequencing libraries were generated using a NEBNext Ultra Library Prep Kit for Illumina (New England Biolabs, Ipswich, MA, USA), following manufacturer’s recommendations. An Agilent Bioanalyzer 2100 system was used to assess library quality. The clustering of index-coded samples was performed on a cBot Cluster Generation System using the PE Cluster cBot-HS Kit (Illumina, San Diego, CA, USA). Subsequent, paired-end, 150 bp sequencing was performed by Novogene. Raw reads were submitted to the NCBI SRA database (accession: PRJNA694644).

### 2.4. Transcriptome Assembly and Functional Annotation

To remove Illumina adapter sequences and filter out poor quality reads from raw read data, fastp (version 0.20.0) was used. All downstream analyses were based on cleaned, trimmed reads. HISAT2 (version 2.0.5) was used to map all pooled, trimmed reads to the rainbow trout reference genome (GCF_002163495.1_Omyk_1.0_genomic.gtf). For novel gene prediction, StringTie (version 1.3.3) and GffCompare (version 0.10.6) were used to assemble the set of transcript isoforms of each bam file obtained from the mapping step and compare StringTie assemblies to the rainbow trout reference annotation file to sort out novel genes from known ones. FeatureCounts (version 1.5.0-p3) was used to count the read numbers that were mapped to each gene. Reads Per Kilobase of exon model per Million mapped reads (RPKM) of each gene was calculated based on gene length and read count. Differential expression quantification was generated using DESeq2 (version 1.20.0) at *p* < 0.05 following Benjamini-Hochberg’s false discovery rate correction (FDR) [35]. ClusterProfiler (version 3.24.3) was used for functional annotation in Gene Ontology (GO) and Kyoto Encyclopedia of Genes and Genomes (KEGG) Pathway enrichment analyses at a significance of FDR < 0.05. Significant, differentially expressed genes (DEGs) were uploaded to Ingenuity Pathway Analysis (IPA; Qiagen; Valencia, CA, USA) to generate predicted canonical, disease and function, and network relationships between treatment groups.

### 2.5. Quantitative Polymerase Chain Reaction (qPCR) Validation of Differentially Expressed Genes

To validate expression profiles of DEGs determined from RNA sequencing analysis, qPCR was conducted on select genes involved in the top predicted pathways altered following bifenthrin treatment. RNA was diluted to 1 µg and reverse transcribed to cDNA using an iScript Reverse Transcription Supermix kit (Bio-Rad, Hercules, CA, USA). To perform qPCR, a 20 µL reaction was performed, with an input concentration of 100 ng cDNA and 10 µM of primer pairs (*csf1*, *cbs*, *ptafr*, and *mtap*; Supplementary Table S1). All qPCR reactions were conducted in triplicate and run on a Bio-Rad CFX Connect Real-Time PCR Detection System and followed the same thermal cycling conditions as previously described [23]. To determine expression fold change between treatment and control groups, 2^−ΔΔCt^ was used [36], with all results normalized to the housekeeping gene, *ef1α*, as no significant difference in expression was observed between treatments.

### 2.6. Histopathological Analysis

Rainbow trout brains were fixed in Bouin’s solution for 24 h, transferred to 70% ethanol, and sent to HistoWiz (Brooklyn, NY, USA) for sectioning and tissue staining. Sagittal sections of each brain, 5 µm sections, were taken in triplicate on each slide, with a total of four biological replicates per exposure treatment (*n* = 4). Each brain section underwent a terminal deoxynucleotidyl transferase dUTP nick end labeling (TUNEL) stain to detect apoptosis from fragmentation of DNA strand breaks. Apoptotic cells were identified by the presence of brown staining, manually counted in the cerebellum and optic tectum regions, and quantified by the number of TUNEL positive cells per 1 mm^2^ area.

## 3. Results

### 3.1. Waterborne Bifenthrin Concentrations

The mean concentrations of bifenthrin in the control treatment group throughout the duration of the exposures were below the reporting limit (<2 ng/L; Supplementary Table S2). The mean ± standard deviation concentration of the nominal 15 ng/L bifenthrin treatment from 24 h, 7 d, and 14 d was 12.82 ± 2.23, 8.78 ± 1.04, and 6.26 ± 1.15 ng/L, respectively. The mean percent DCBP surrogate recovery was 86.74 ± 4.16, 40.55 ± 6.49, and 21.04 ± 0.65%, respectively. The mean concentration of the nominal 30 ng/L bifenthrin treatment from 24 h, 7 d, and 14 d was 21.68 ± 0.004, 30.66 ± 7.72, and 12.88 ± 1.94 ng/L, respectively. The mean percent DCBP surrogate recovery was 90.21 ± 0.61, 60.9 ± 17.73, and 18.05 ± 4.37%, respectively (Supplementary Table S2).

### 3.2. Transcriptome Assembly and Annotation

**Figure 1:**
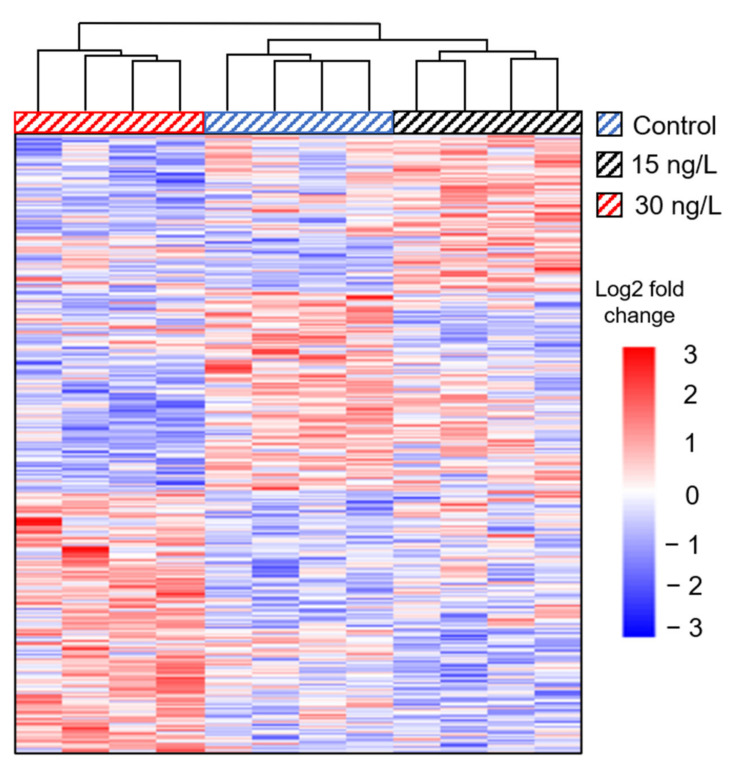
Heatmap dendrogram comparing differentially expressed genes (DEGs) in the brains of juvenile rainbow trout exposed to 0, 15, and 30 ng/L bifenthrin.

A total of 530,790,834 mapped reads were generated from rainbow trout, with 306,265,598 bases assembled following adapter trimming (~25.5 million/replicate) and used in transcriptome assembly (Supplementary Table S3). More than 86% of the reads aligned to the transcriptome using HISAT2, indicating a representative transcriptome assembly. There were 21 significantly, differentially expressed genes (DEGs) in the 15 ng/L bifenthrin treatment group and 131 DEGs in the 30 ng/L bifenthrin treatment group, relative to controls (FDR < 0.05; Supplementary Figure S1). Additionally, there were 43 DEGs between the 15 and 30 ng/L treatment groups. A heatmap dendrogram depicts DEGs strongly clustered between treatment groups (Figure 1).

**Figure 2:**
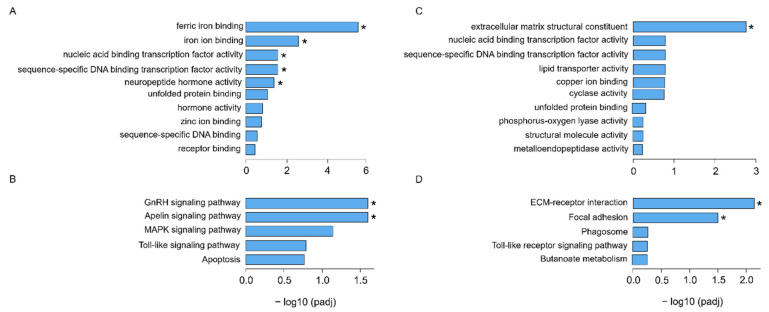
Top GO molecular function (MF) pathways in the brains of juvenile rainbow trout exposed to (**A**) 15 ng/L and (**C**) 30 ng/L bifenthrin, and KEGG pathways from trout exposed to (**B**) 15 ng/L and (**D**) 30 ng/L bifenthrin. The asterisks denote statistically significantly differences between exposure treatment groups and controls (FDR < 0.05).

Gene ontology (GO) molecular processes involved in ferric iron binding (*ferritin*), iron binding (*ferritin*), and nucleic acid binding transcription (*transcription factor jun-D, nuclear hormone receptor HR38, proto-oncogene c-Fos*), sequence-specific DNA binding transcription (*transcription factor jun-D, nuclear hormone receptor HR38, proto-oncogene c-Fos*), and neuropeptide hormone activity (*isotocin-neurophysin IT 2*) were significantly altered in trout exposed to 15 ng/L bifenthrin, relative to controls (Figure 2A). Homeostatic pathways including cellular iron ion, iron ion, cellular cation, and chemical homeostasis were among the top ten altered GO biological function pathways in trout exposed to 15 ng/L bifenthrin (Supplementary Table S4). Within the top five KEGG pathways, GnRH and apelin signaling pathways were significantly dysregulated in trout exposed to 15 ng/L bifenthrin (Figure 2B, Supplementary Table S5). The extracellular matrix structural constituent (*collagen alpha-1(I) chain*), nucleic acid binding transcription factor, sequence-specific DNA binding transcription, and lipid transporter activity pathways were among the top GO molecular processes altered following exposure to 30 ng/L bifenthrin (Figure 2A, Supplementary Table S4), though ECM structural constituent was the only significantly altered GO pathway following treatment (Figure 2C, Supplementary Table S4). Similarly, ECM-receptor interaction and focal adhesion were the top two KEGG pathways significantly altered in rainbow trout exposed to 30 ng/L bifenthrin, relative to controls (Figure 2D, Supplementary Table S5).

The top diseases and functions predicted in IPA in rainbow trout exposed to 15 ng/L were involved in cell-to-cell signaling and interaction, cellular compromise, organismal injury and abnormalities, cellular development, and cell death and survival (Supplementary Table S6). Among the top 10 predicted canonical pathways affected in the low, 15 ng/L treatment group were associated with *S*-methyl-5′-thioadenosine degradation II, histidine degradation III and IV, and coagulation system (Supplementary Figure S2). The top altered network was involved in cell-to-cell signaling and interaction, cellular movement, and immune cell trafficking, with a score of 22 and 9 DEGs involved (Supplementary Figure S3). Organismal injury and abnormalities, tissue morphology, hematological disease, molecular transport, cell-to-cell signaling and interaction, and reproductive system development and function were among the top 15 diseases and functions predicted to be altered in the brains of rainbow trout exposed to 30 ng/L (Supplementary Table S6). The top predicted canonical pathways in the 30 ng/L treatment group were involved in *S*-methyl-5′-thioadenosine degradation II, cysteine biosynthesis/homocysteine degradation, neuroprotective role of THOP1 in Alzheimer’s disease, extrinsic prothrombin activation pathway, and coagulation system (Supplementary Figure S2). The top altered network was involved in cell cycle, cell-to-cell signaling and interaction, and cellular compromise, with a score of 29 and 12 DEGs involved (Supplementary Figure S4).

### 3.3. qPCR Validation

A subset of genes (*csf1*, *cbs*, *ptafr*, and *mtap*) determined to be differentially expressed following RNA sequencing were verified by qPCR. The expression fold change of *csf1* (1.19), *cbs* (1.11), *ptafr* (1.96), and *mtap* (−1.01) following RNA sequencing exhibited similar fold change expression relationships determined by qPCR, *csf1* (1.26), *cbs* (1.16), *ptafr* (1.49), and *mtap* (−0.90), which confirmed expression patterns (Supplementary Figure S5).

### 3.4. TUNEL Positive, Apoptotic Cell Assessment

**Figure 3:**
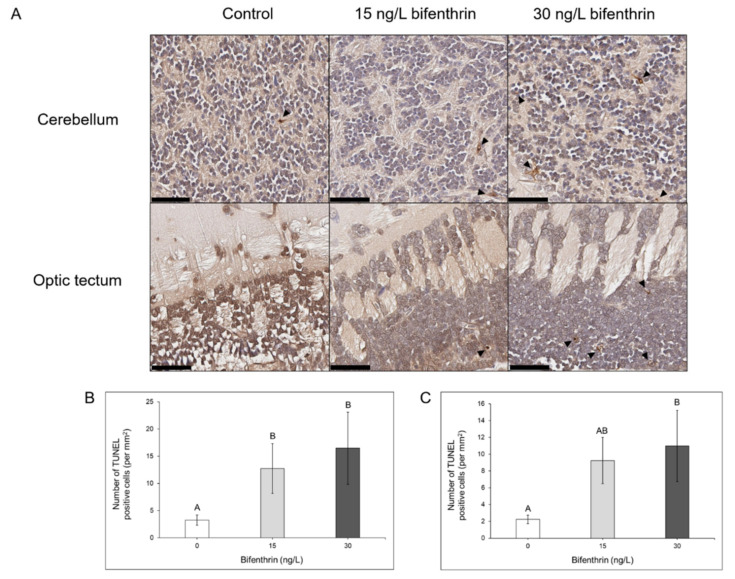
TUNEL positive cells assessed in the (**A**,**B**) cerebellum and (**A**,**C**) optic tectum of juvenile rainbow trout exposed to 0, 15, and 30 ng/L bifenthrin. TUNEL positive cells were quantified by the number of brown-stained nuclei per 1 mm^2^, denoted by dark arrows, and black bars represent a 50 µM scale bar (**A**). Treatments with different uppercase letters are representative of statistically significant treatments from other treatment groups (*p* < 0.05).

Rainbow trout exposed to 15 and 30 ng/L bifenthrin had significantly increased numbers of TUNEL positive, apoptotic cells in the cerebellum, relative to control (*p* = 0.045 and *p* = 0.008, respectively; Figure 3A,B). Trout exposed to 30 ng/L bifenthrin exhibited a significantly increased number of apoptotic cells in the optic tectum (*p* = 0.026; Figure 3A,C), though not in trout exposed to 15 ng/L bifenthrin (*p* = 0.100; Figure 3C).

## 4. Discussion

Transcriptomic profiles were determined in the brains of rainbow trout, a surrogate to the critically endangered steelhead trout in California, following a two-week bifenthrin treatment to concentrations frequently measured within water samples (≤30 ng/L) from the Sacramento-San Joaquin Delta. Significantly altered DEGs were incorporated into multiple functional annotation tools (GO, KEGG, and IPA), with genes involved in the top predicted pathways further validated using qPCR. Histopathological assessment was conducted in the brain and TUNEL-positive cells were identified in the cerebellum and optic tectum of exposed fish, as apoptotic-associated pathways were recently shown to be the top predicted pathways impaired in both steelhead trout and Chinook salmon following bifenthrin treatment for two-week and 96 h durations, respectively [23,24], using non-targeted metabolomics.

KEGG pathway analyses involved in GnRH signaling were the top significantly altered functional pathways impaired in the brains of trout exposed to 15 ng/L bifenthrin (*p*-adj = 2.55 × 10^−2^), based on the decrease of *egr1*. *Egr1*, early growth response protein 1, is within the immediate early gene (IEG) family [37] and considered a primary response gene due to its rapid response to changes in GnRH expression [38,39], with a positive increased correlation between the induction of *egr1* expression relative to the concentration of GnRH [39]. The dysregulation of *GnRH* expression has previously been observed in the brains of rainbow trout following a 96 h and two-week exposure to 1.5 and 0.15 µg/L bifenthrin, respectively [20], as well as in Chinook salmon exposed to 1.5 µg/L bifenthrin for 96 h [21]. Our results indicate that altered expression changes of genes involved in the dopaminergic pathway are impaired in salmonids even at low ng/L concentrations following bifenthrin exposure and suggests that changes to this pathway are among the most sensitive to juvenile salmonids.

The top GO molecular function pathways affected in the 15 ng/L bifenthrin treatment were related to ferric iron binding (*p*-adj = 2.48 × 10^−6^), based on the upregulation of *ferritin*, and nucleic acid binding transcription factor activity (*p*-adj = 2.50 × 10^−2^), based on the dysregulation of *jun-D*, *HR38*, and *c-Fos*. Ferritin is an iron storage protein that is predominantly expressed within oligodendrocytes, astrocytes, and microglia in the brain, with a predominant role in the intracellular homeostatic balance of iron [40,41]. Improper mRNA *ferritin* expression can induce oxidative stress, induce inflammatory responses, and impact cell survival [42,43].

The expression of *jun-D*, a dysregulated gene involved in proper nucleic acid binding transcription in rainbow trout exposed to bifenthrin, also serves as a transcriptional regulator of ferritin from electrophilic xenobiotics and oxidative stress [43] and was significantly increased in trout following 15 ng/L bifenthrin exposure. Oxidative stress, respiration, and iron binding functional annotation pathways comprised 6% of the DEGs in juvenile delta smelt (*Hypomesus transpacificus*) exposed to 0.0625 and 0.125 µg/L esfenvalerate [44] and cellular iron ion homeostasis was among the top GO pathways affected in larval delta smelt exposed to 4.84 µg/L permethrin for 96 h [45]. Furthermore, altered iron homeostasis can induce oxidative stress and generate reactive oxygen species [46], which was recently shown to be involved in the inflammatory pathway of steelhead exposed to 120 ng/L bifenthrin [23]. This suggests that dysregulated iron homeostatic pathways may be a general indicator of pyrethroid exposure [45] and subsequently impair neuronal cell structure.

The extracellular matrix structural constituent GO pathway was significantly altered in trout exposed to 30 ng/L bifenthrin (*p*-adj = 1.72 × 10^−3^), based on the dysregulation of *col1a1*, *col1a2*, and *col1a3*. The expression of two of these extracellular matrix structural collagens, *col1a2* and *col1a1*, were previously reported to be inhibited in larval delta smelt exposed to 4.84 µg/L permethrin for 96 h [45]. Interestingly, a transcriptomic and proteomic-based study conducted on the hypothalamus of largemouth bass (*Micropterus salmoides*) following a dietary exposure to 3 mg dieldrin/kg noted changes in *col1a1* mRNA expression, as well as Col1a protein levels [47]. Impaired structural integrity of the extracellular matrix and associated proteins that play important roles in cell structure may induce underlying apoptotic effects in the brains of fish following pyrethroid exposure, as previously suggested with dieldrin [47].

Cell-to-cell signaling, cellular compromise, and cell death and survival were among the top predicted disease and function pathways impaired in the brains of rainbow trout exposed to 15 and 30 ng/L bifenthrin, determined by IPA, further supporting altered GO pathways. The functional annotations were related to impaired cell attachment, disorganization of cytoskeleton, and apoptosis of macrophage precursor cells. Juvenile steelhead exposed to higher concentrations of bifenthrin, 60 and 120 ng/L, were recently shown to have altered molecular and cellular functions including cellular compromise and cell signaling pathways, respectively [23]. Impacts to GO biological processes related to cellular signaling were impaired in embryonic zebrafish exposed to 40 µg/L deltamethrin [48], with top pathways involved in neuronal systems and cell-to-cell adhesion and signaling. Additionally, altered expression of a cell adhesion protein found in the extracellular matrix was dysregulated in larval delta smelt exposed to <2 µg/L permethrin [45]. These findings support the top altered KEGG pathways in rainbow trout exposed to 30 ng/L bifenthrin, with impaired focal adhesion, suggesting a potential relationship between downstream effects of dysregulated iron homeostatic pathways and structural integrity of cell attachment sites and extracellular matrix, though further study is needed to support this interrelationship and influence on apoptotic alterations.

The top predicted disease and function pathways in IPA included cell death and survival in bifenthrin-exposed rainbow trout. Similar pathways were previously observed in the brains of juvenile steelhead treated with 60 ng/L bifenthrin [23] and Chinook exposed to 150 ng/L bifenthrin [24] when assessed for metabolomic profile changes. There was a significantly increased incidence of TUNEL positive cells in the cerebellum of 15 and 30 ng/L bifenthrin-exposed rainbow trout and a significant increase in the optic tectum of 30 ng/L bifenthrin-exposed trout, which validates predicted metabolomic [23,24] and transcriptomic-level apoptotic pathways. The endangered fish mahseer (*Tor putitora*) exposed to 63 µg/L cypermethrin for 96 h [49] and silver carp (*Hypophthalmichtys molitrix*) exposed to 2 µg/L deltamethrin for 96 h [50] also exhibited histopathological changes in the brain, including observed neuronal degeneration, infiltration, and spongiosis. However, this is the first known study that quantitatively assessed neuronal apoptotic histopathological effects in fish following bifenthrin treatment.

Dysregulated pathways involved in maintaining structural stability of the extracellular matrix and impaired focal adhesion in the brains of bifenthrin-exposed trout are consistent with oxidative stress induced effects previously observed in neuronal in vitro cell lines and in murine, in vivo-based studies, following bifenthrin treatment [51,52,53,54], though, interestingly, oxidative stress genes were not differentially altered in bifenthrin treatment groups, relative to controls. Similarly, we previously reported that steelhead trout exposed to 60 and 120 ng/L bifenthrin were predicted to have induced reactive oxygen species production due to oxidative stress, and subsequently observed apoptotic effects driven by altered levels of metabolites [23]. Increased apoptotic cells were observed in bifenthrin-exposed trout, determined histopathologically, and KEGG predicted apoptotic pathways at *p* = 0.057 in rainbow trout exposed to 30 ng/L bifenthrin in the current transcriptomics-based study. It is possible that the predicted ECM and apoptotic pathways occurred as an acute phase response, which may explain the relatively weak transcriptomic relationship due to the duration of the response. Overall, there were relatively few differentially expressed genes following annotation relative to the number of altered metabolites previously identified [23,24], which has also been observed by others [55]. Additionally, concentrations of bifenthrin that steelhead were previously exposed to, 60 and 120 ng/L, were likely high enough to induce transcriptomic level changes from altered metabolite levels. Furthermore, the steelhead previously assessed were significantly smaller than the current rainbow trout, which could influence the sensitivity to bifenthrin, as well as the rate of metabolism. To better characterize the mechanistic effects of bifenthrin, combining several omic-level responses within the same species and life stage, while utilizing the same exposure concentrations, would be beneficial to understand the interrelationship between molecular, histological, and behavioral-level effects.

## 5. Conclusions

Juvenile rainbow trout exposed to environmentally relevant concentrations of bifenthrin exhibited transcriptomic-level changes and histopathological alterations throughout the cerebellum and optic tectum, as determined by increased TUNEL positive cells. This is the first known study to report neurotoxic effects of bifenthrin at low ng/L concentrations and subsequent histopathological insult to brain structure. Additional studies focused on region-specific areas of the brain where dysregulated iron homeostasis and impaired cell structure are predominantly occurring are warranted to better characterize the neurotoxic effect of bifenthrin. Furthermore, integrating multiple ‘omic-based approaches using functional annotation networks would provide a clearer depiction of bifenthrin’s mode of action and the influence of species, life stage, and concentration to neurotoxic responses, which could be used in ecological risk assessment decisions.

## Data Availability

The data presented in this study are available on request from the corresponding author.

## Supplementary Materials

The following are available online at https://www.mdpi.com/2305-6304/9/3/48/s1, Figure S1: Volcano plots of differentially expressed genes (DEGs) between control and 15 ng/L (A), control and 30 ng/L (B), and 15 and 30 ng/L bifenthrin (C) treatment groups. Upregulated genes are depicted in red, downregulated genes depicted in green, and those not significanlty different depicted in blue (FDR < 0.05). Figure S2: Top predicted Ingenuity Pathway Analysis (IPA) canonical pathways for juvenile rainbow trout treated with (A) 15 and (B) 20 ng/L bifenthrin. Figure S3: Top predicted Ingenuity Pathway Analysis (IPA) network involved in cell-to-cell signaling and interaction, cellular movement, and immune cell trafficking in juvenile rainbow trout exposed to 15 ng/L bifenthrin. Figure S4: Top predicted Ingenuity Pathway Analysis (IPA) network involved in cell cycle, cell-to-cell signaling and interaction, and cellular compromise in juvenile rainbow trout exposed to 30 ng/L bifenthrin. Figure S5: Comparison between differentially expressed genes determined by RNA sequencing and qPCR expression fold change patterns in the brains of bifenthrin-exposed rainbow trout (mean ± SD, One-way ANOVA, Tukey’s post hoc (*p* < 0.05)). Table S1: Primer pair sequences used for qPCR validation. Table S2: Mean bifenthrin concentration ± standard deviation (SD) in extract following a 24 hour (h), 7 day (d), and 14 d treatment. Mean percent recoveries are based on decachlorobiphenyl surrogate standards. Corrected mean bifenthrin concentrations in parentheses. Table S3: Total number of raw and clean RNA sequencing reads. Table S4: Top 10 gene ontology (GO) biological pathways (BP) and molecular functions (MF) impaired in juvenile rainbow trout exposed to 15 and 30 ng/L bifenthrin. Table S5: Top 5 KEGG pathways impaired in juvenile rainbow trout exposed to 15 and 30 ng/L bifenthrin. Table S6: Top 20 diseases and functions predicted in Ingenuity Pathway Analysis in rainbow trout exposed to 15 and 30 ng/L bifenthrin.
