# Supplementary material for: Transcriptomic and Histopathological Effects of Bifenthrin to the Brain of Juvenile Rainbow Trout (Oncorhynchus mykiss)
Source: Toxics. 2021 Mar 5;9(3):48. doi: 10.3390/toxics9030048 (PMC8000926; doi:10.3390/toxics9030048)
Supplement: Supplementary file 1 [file toxics-09-00048-s001.pdf]

Article

Transcriptomic and Histopathological Effects of Bifenthrin to the Brain of Juvenile Rainbow Trout (*Oncorhynchus mykiss*)

Jason T. Magnuson, Kara E. Huff Hartz, Corie A. Fulton, Michael J. Lydy and Daniel Schlenk

Table S1. Primer pair sequences used for qPCR validation.

Gene	Direction	Sequence	Amplicon Length	Efficiency (%)	Accession Number
csf1	Forward	CCTTCACCCAATGGAACTGAGTC	78	90.8	XM_036947884.1
	Reverse	CGTCTGCCACGAATGAACCT			
cbs	Forward	GGATAGGATCAGTCTGCGTATGGTG	110	90.0	NM_001124686.1
	Reverse	GCAATACAGGTATCGGTCTGGCTC			
ptafr	Forward	CTTATCATTGTGGGGTGCTTCGTC	96	88.4	XM_021584417.2
	Reverse	TACTAGCCCAGTCCCTTACCCAG			
mtap	Forward	CCATCATGCCAACCGACGT	78	93.7	NM_001160570.1
	Reverse	CTGCACACATCTCCTGGTCA			
ef1α	Forward	GAGAACCATTGAAAAGTTCGAGAAG	71	91.4	XM_021561980.1
	Reverse	GCACCCAGGCATACTTGAAAG			

Table S2. Mean bifenthrin concentration ± standard deviation (SD) in extract following a 24 hour (h), 7 day (d), and 14 d treatment. Mean percent recoveries are based on decachlorobiphenyl surrogate standards. Corrected mean bifenthrin concentrations in parentheses.

Nominal Bifenthrin Concentration (ng/L)	Mean 24 h (ng/L)	Mean 24 h Recovery (%)	Mean 7 d (ng/L)	Mean 7 d Recovery (%)	Mean 14 d (ng/L)	Mean 14 d Recovery (%)
0	< BRL	86.89 ± 2.70	< BRL	32.75 ± 18.31	< BRL	36.36 ± 10.55
15	11.36 ± 2.39 (12.82)	86.74 ± 4.16	5.50 ± 0.43 (8.78)	40.55 ± 6.49	3.50 ± 0.63 (6.26)	21.04 ± 0.65
30	19.75 ± 0.11 (21.68)	90.21 ± 0.61	22.58 ± 8.43 (30.66)	60.9 ± 17.73	7.10 ± 1.24 (12.88)	18.05 ± 4.37

BRL, Below reporting limit.

Table S3. Total number of raw and clean RNA sequencing reads.

Sample	Total Raw Reads	Total Clean Reads	Clean Reads Q20 (%)	Clean Reads Q30 (%)	GC Content (%)
Control 1	28526973	27648230	97.59	93.49	47.49
Control 2	25532536	24524205	97.76	93.93	47.00
Control 3	26102740	25419205	97.82	94.05	47.94
Control 4	28596217	27447141	97.73	93.85	47.47
15 ng/L bifenthrin 1	27416655	26453013	97.74	93.90	47.33
15 ng/L bifenthrin 2	29876619	29070074	97.55	93.42	47.72
15 ng/L bifenthrin 3	26466053	25427697	97.76	93.96	47.13
15 ng/L bifenthrin 4	28003792	27033403	97.59	93.55	47.43
30 ng/L bifenthrin 1	29355288	28305590	97.51	93.41	47.21
30 ng/L bifenthrin 2	22426419	21954219	97.56	93.46	47.61
30 ng/L bifenthrin 3	21877612	21212383	98.04	94.84	47.79
30 ng/L bifenthrin 4	22243920	21770438	98.10	95.03	47.61

Table S4. Top 10 gene ontology (GO) biological pathways (BP) and molecular functions (MF) impaired in juvenile rainbow trout exposed to 15 and 30 ng/L bifenthrin.

Treatment	GO Category	ID	Description	p-Value	FDR
15 ng/L					
	BP	GO:0006879	cellular iron ion homeostasis	2.50×10^{-7}	1.75×10^{-6}
		GO:0055072	iron ion homeostasis	2.50×10^{-7}	1.75×10^{-6}
		GO:0006873	cellular ion homeostasis	1.03×10^{-6}	1.91×10^{-6}
		GO:0006875	cellular metal ion homeostasis	1.03×10^{-6}	1.91×10^{-6}
		GO:0030003	cellular cation homeostasis	1.03×10^{-6}	1.91×10^{-6}
		GO:0055065	metal ion homeostasis	1.03×10^{-6}	1.91×10^{-6}
		GO:0055082	cellular chemical homeostasis	1.03×10^{-6}	1.91×10^{-6}
		GO:0050801	ion homeostasis	1.23×10^{-6}	1.91×10^{-6}
		GO:0055080	cation homeostasis	1.23×10^{-6}	1.91×10^{-6}
		GO:0048878	chemical homeostasis	1.46×10^{-6}	2.04×10^{-6}
	MF	GO:0008199	ferric iron binding	2.25×10^{-7}	2.48×10^{-6}
		GO:0005506	iron ion binding	4.16×10^{-4}	2.29×10^{-3}
		GO:0001071	nucleic acid binding transcription factor activity	9.10×10^{-3}	2.50×10^{-2}
		GO:0003700	sequence-specific DNA binding transcription factor activity	9.10×10^{-3}	2.50×10^{-2}
		GO:0005184	neuropeptide hormone activity	1.72×10^{-2}	3.79×10^{-2}
		GO:0051082	unfolded protein binding	4.31×10^{-2}	7.91×10^{-2}
		GO:0005179	hormone activity	8.95×10^{-2}	1.41×10^{-1}
		GO:0008270	zinc ion binding	1.17×10^{-1}	1.61×10^{-1}
		GO:0043565	sequence-specific DNA binding	2.09×10^{-1}	2.55×10^{-1}
		GO:0005102	receptor binding	3.07×10^{-1}	3.37×10^{-1}
30 ng/L					
	BP	GO:0006869	lipid transport	7.22×10^{-3}	3.95×10^{-1}
		GO:0010876	lipid localization	7.22×10^{-3}	3.95×10^{-1}
		GO:0071702	organic substance transport	9.79×10^{-3}	3.95×10^{-1}
		GO:0006457	protein folding	3.12×10^{-2}	5.30×10^{-1}
		GO:0009190	cyclic nucleotide biosynthetic process	3.32×10^{-2}	5.30×10^{-1}
		GO:0009187	cyclic nucleotide metabolic process	3.52×10^{-2}	5.30×10^{-1}
		GO:0006164	purine nucleotide biosynthetic process	4.59×10^{-2}	5.30×10^{-1}
		GO:0072522	purine-containing compound biosynthetic process	5.28×10^{-2}	5.30×10^{-1}
		GO:0006325	chromatin organization	7.29×10^{-2}	5.30×10^{-1}
		GO:0016571	histone methylation	9.76×10^{-2}	5.30×10^{-1}
	MF	GO:0005201	extracellular matrix structural constituent	1.42×10^{-5}	1.72×10^{-3}
		GO:0001071	nucleic acid binding transcription factor activity	4.24×10^{-3}	1.65×10^{-1}
		GO:0003700	sequence-specific DNA binding transcription factor activity	4.24×10^{-3}	1.65×10^{-1}
		GO:0005319	lipid transporter activity	7.07×10^{-3}	1.65×10^{-1}
		GO:0005507	copper ion binding	8.19×10^{-3}	1.65×10^{-1}
		GO:0009975	cyclase activity	8.19×10^{-3}	1.65×10^{-1}
		GO:0051082	unfolded protein binding	2.71×10^{-2}	4.68×10^{-1}
		GO:0016849	phosphorus-oxygen lyase activity	3.75×10^{-2}	5.54×10^{-1}

	GO:0005198	structural molecule activity	4.12×10^{-2}	5.54×10^{-1}
	GO:0004222	metalloendopeptidase activity	4.92×10^{-2}	5.92×10^{-1}

Table S5. Top 5 KEGG pathways impaired in juvenile rainbow trout exposed to 15 and 30 ng/L bifenthrin.

Treatment	KEGG ID	Description	*p*-Value	FDR
15 ng/L				
	otw04912	GnRH signaling pathway	4.06×10^{-3}	2.55×10^{-2}
	otw04371	Apelin signaling pathway	7.29×10^{-3}	2.55×10^{-2}
	otw04010	MAPK signaling pathway	3.12×10^{-2}	7.28×10^{-2}
	otw04620	Toll-like receptor signaling pathway	9.22×10^{-2}	1.61×10^{-1}
	otw04210	Apoptosis	1.37×10^{-1}	1.73×10^{-1}
30 ng/L				
	otw04512	ECM-receptor interaction	1.69×10^{-4}	7.08×10^{-3}
	otw04510	Focal adhesion	1.48×10^{-3}	3.11×10^{-2}
	otw04145	Phagosome	4.30×10^{-2}	5.40×10^{-1}
	otw04620	Toll-like receptor signaling pathway	8.22×10^{-2}	5.40×10^{-1}
	otw00650	Butanoate metabolism	9.29×10^{-2}	5.40×10^{-1}

Table S6. Top 20 diseases and functions predicted in Ingenuity Pathway Analysis in rainbow trout exposed to 15 and 30 ng/L bifenthrin.

Treatment	Category	Diseases or Functions Annotation	*p*-Value
15 ng/L			
	Cell-To-Cell Signaling and Interaction	Attachment of cells	6.86×10^{-6}
	Cellular Compromise	Disorganization of cytoskeleton	7.99×10^{-5}
	Organismal Injury and Abnormalities	Formation of scar tissue	8.99×10^{-5}
	Cancer	Proliferation of tumor cells	1.31×10^{-4}
	Cellular Development	Colony formation of phagocytes	1.31×10^{-4}
	Cellular Growth and Proliferation	Colony formation of phagocytes	1.31×10^{-4}
	Tumor Morphology	Proliferation of tumor cells	1.31×10^{-4}
	Cell Death and Survival	Apoptosis of macrophage precursor cells	1.76×10^{-4}
	Connective Tissue Development and Function	Survival of osteoclasts	1.76×10^{-4}
	Skeletal and Muscular System Development and Function	Survival of osteoclasts	1.76×10^{-4}
	Hematological Disease	Infection of myeloid cells	2.89×10^{-4}
	Infectious Diseases	Infection of myeloid cells	2.89×10^{-4}
	Hematological System Development and Function	Colony formation of phagocytes	3.47×10^{-4}
	Hematopoiesis	Colony formation of phagocytes	3.47×10^{-4}
	Lymphoid Tissue Structure and Development	Colony formation of phagocytes	3.47×10^{-4}
	Tissue Development	Colony formation of phagocytes	3.47×10^{-4}
	Cellular Function and Maintenance	Macropinocytosis	4.54×10^{-4}
	Amino Acid Metabolism	Deamination of L-lysine	7.92×10^{-4}
	Cardiovascular Disease	Familial thoracic aortic aneurysm type 10	7.92×10^{-4}
	Cell Cycle	Replication of epidermal cells	7.92×10^{-4}
30 ng/L			
	Cardiovascular Disease	Abnormal morphology of blood vessel	6.42×10^{-5}
	Cardiovascular System Development and Function	Abnormal morphology of blood vessel	6.42×10^{-5}
	Organismal Injury and Abnormalities	Abnormal morphology of blood vessel	6.42×10^{-5}

Tissue Morphology	Abnormal morphology of blood vessel	6.42×10^{-5}
Hematological Disease	Hereditary thrombophilia	7.02×10^{-5}
Hereditary Disorder	Hereditary thrombophilia	7.02×10^{-5}
Molecular Transport	Accumulation of cyclic GMP	1.42×10^{-4}
Nucleic Acid Metabolism	Accumulation of cyclic GMP	1.42×10^{-4}
Small Molecule Biochemistry	Accumulation of cyclic GMP	1.42×10^{-4}
Cell-To-Cell Signaling and Interaction	Adhesion of bacteria	1.63×10^{-4}
Reproductive System Development and Function	Regulation of reproductive system	2.11×10^{-4}
Cellular Movement	Cell movement of muscle cells	2.26×10^{-4}
Developmental Disorder	Growth failure or short stature	3.09×10^{-4}
Post-Translational Modification	Hydrolysis of protein fragment	4.19×10^{-4}
Protein Degradation	Hydrolysis of protein fragment	4.19×10^{-4}
Protein Synthesis	Hydrolysis of protein fragment	4.19×10^{-4}
Organismal Development	Abnormal morphology of limb	5.65×10^{-4}
Skeletal and Muscular Disorders	Abnormal morphology of limb	5.65×10^{-4}
Skeletal and Muscular System Development and Function	Abnormal morphology of limb	5.65×10^{-4}
Cell Death and Survival	Purpura fulminans	6.77×10^{-4}

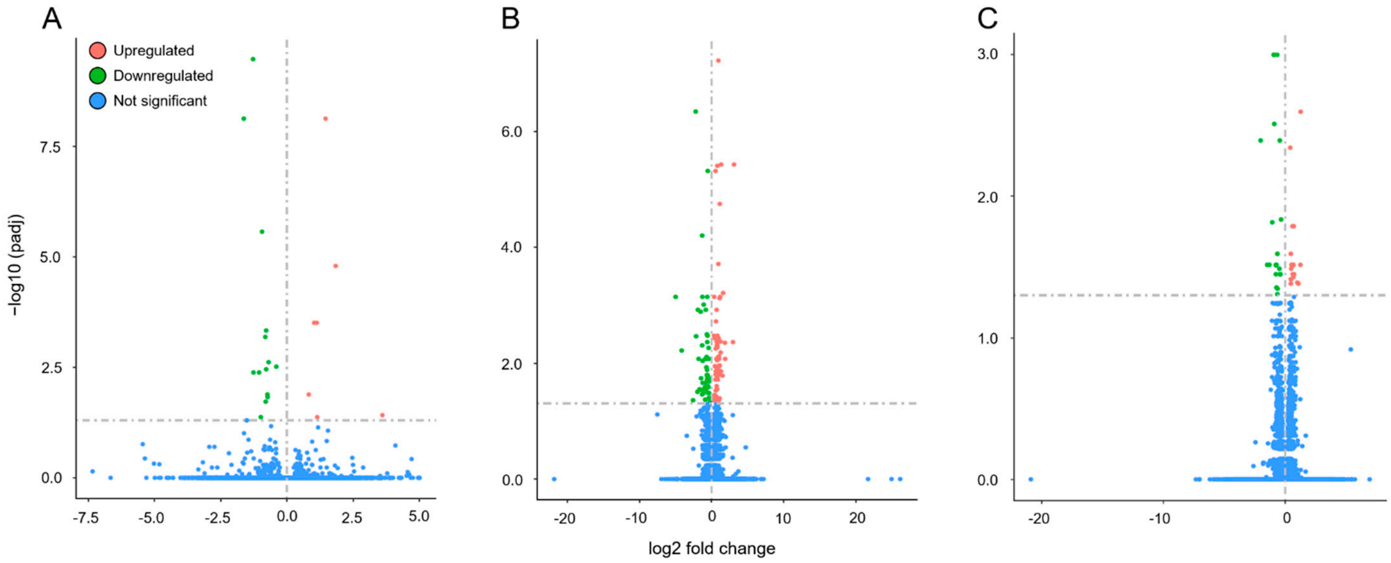

Figure S1. Volcano plots of differentially expressed genes (DEGs) between control and 15 ng/L (**A**), control and 30 ng/L (**B**), and 15 and 30 ng/L bifenthrin (**C**) treatment groups. Upregulated genes are depicted in red, downregulated genes depicted in green, and those not significanlty different depicted in blue (FDR < 0.05).

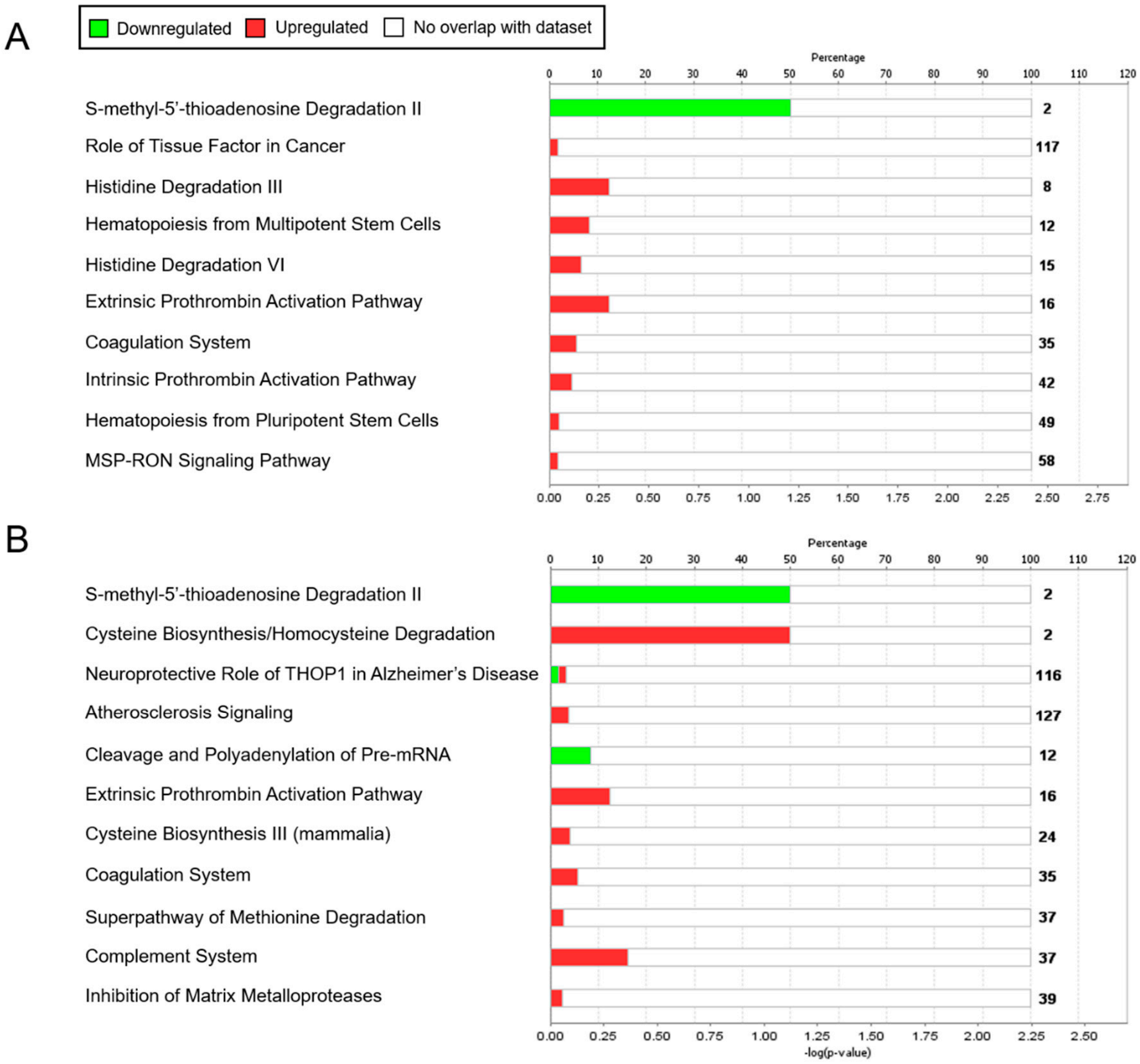

Figure S2. Top predicted Ingenuity Pathway Analysis (IPA) canonical pathways for juvenile rainbow trout treated with (**A**) 15 and (**B**) 20 ng/L bifenthrin.

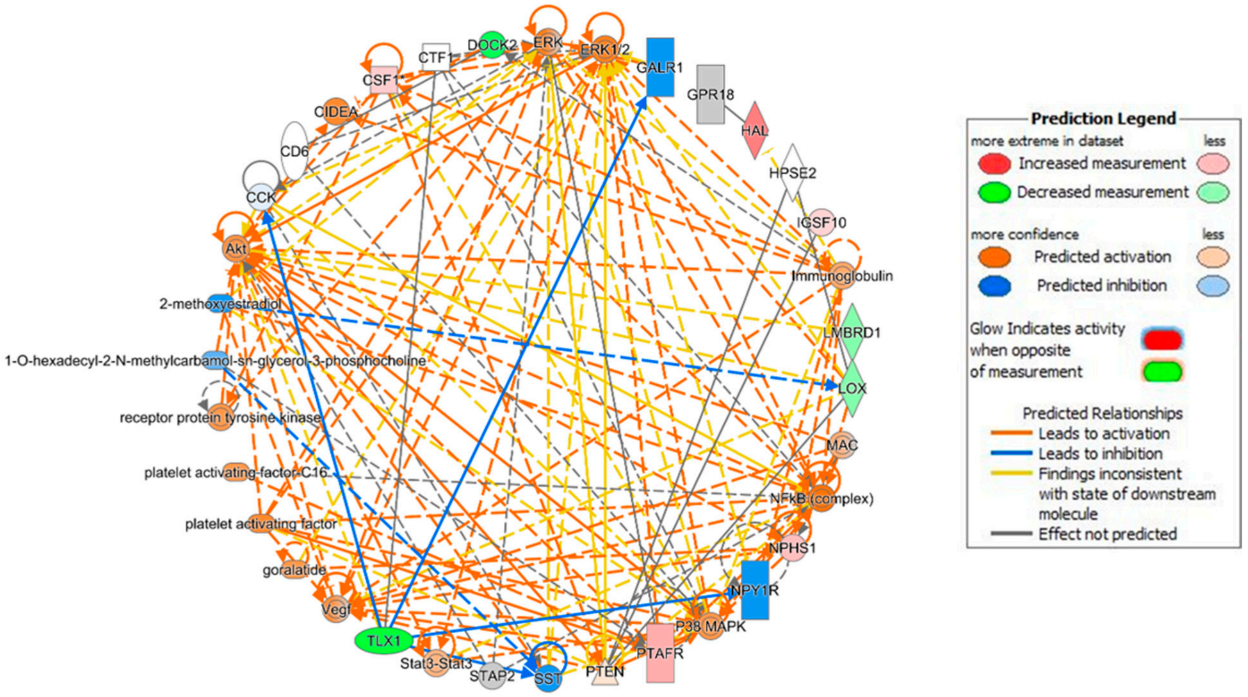

Figure S3. Top predicted Ingenuity Pathway Analysis (IPA) network involved in cell-to-cell signaling and interaction, cellular movement, and immune cell trafficking in juvenile rainbow trout exposed to 15 ng/L bifenthrin.

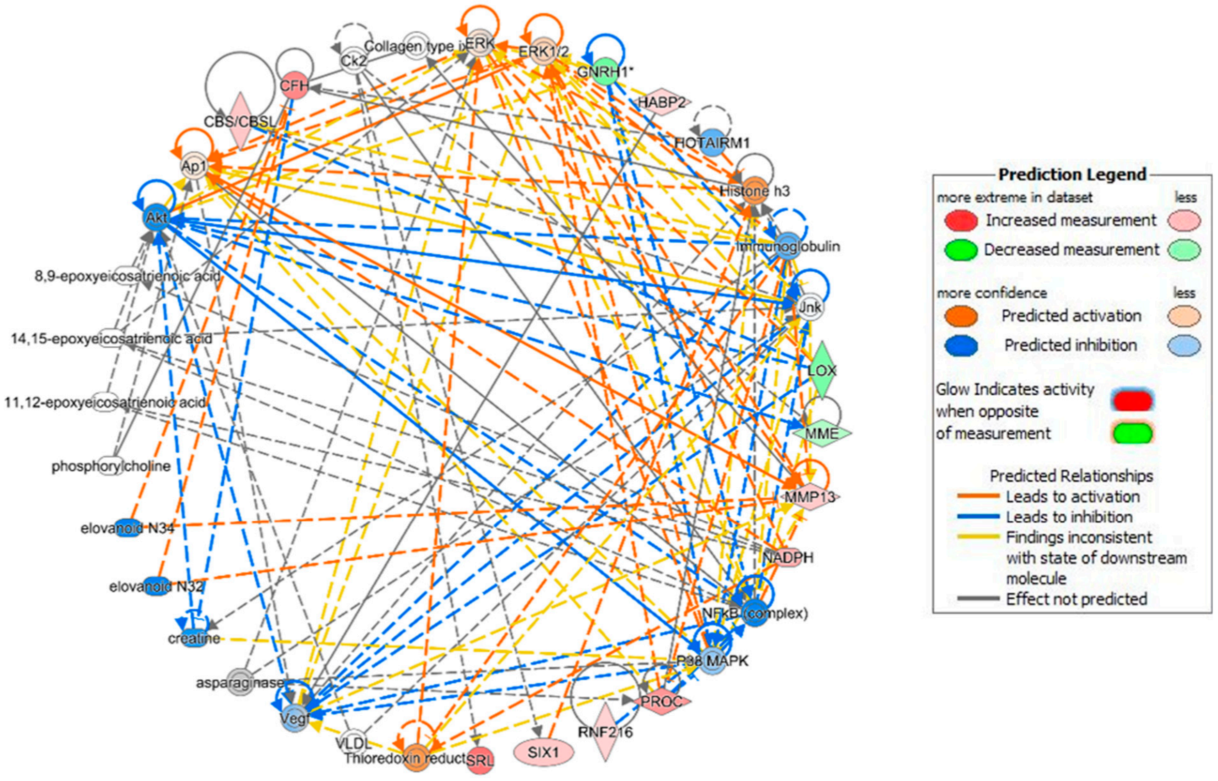

Figure S4. Top predicted Ingenuity Pathway Analysis (IPA) network involved in cell cycle, cell-to-cell signaling and interaction, and cellular compromise in juvenile rainbow trout exposed to 30 ng/L bifenthrin.

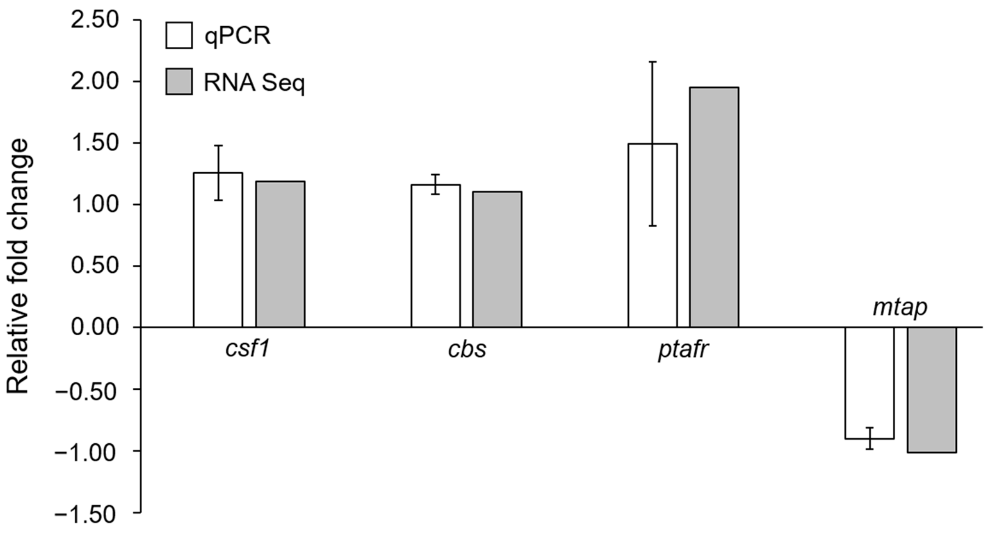

Figure S5. Comparison between differentially expressed genes determined by RNA sequencing and qPCR expression fold change patterns in the brains of bifenthrin-exposed rainbow trout (mean ± SD, One-way ANOVA, Tukey's post hoc ($p <$ 0.05)).
